# Practical Technic of the Bacteriological Diagnosis of Typhoid Fever through Hemoculture

**Published:** 1919

**Authors:** 


**Practical Technic of the Bacteriological Diagnosis of Typhoid Fever through Hemoculture.** By L. TRIBONDEAU. *Paris Médical,* Oct. 26, 1918.

Vaccination has, since the war, completely altered the bacteriological diagnosis of typhoid fever. Serodiagnosis has been largely replaced by hemoculture. It is a sure means of diagnosis and has the immense advantage of giving positive indications at the very onset of the infection, whereas specific agglutinins are traceable at the end of a week in serum.

The execution of this method is simple and speedy, and within the reach of even the most modestly equipped laboratory.

Concerning specimens, the blood must be collected with absolute asepsis. This is fundamental, since the presence of even one foreign germ would vitiate the results of the analysis. The vein puncture is the best method. The skin is first painted with iodine. The quantity of blood to be drawn (from 5 to 10 cc.) is taken directly in a medium tube in glucosed bile provided with a glass agitator. Once the blood is collected, the tube is closed and revolved until the blood is mixed with the bile medium. This bile medium is obtained from one or several biliary vesicles removed from freshly killed oxen, to which are added 1 : 100 peptone and 1 : 100 glucose. This is autoclaved for 20 minutes at 120° C., then shaken and filtered very hot on Chardin paper and finally closed with cotton-wool. Blood is drawn directly into the tube and placed in a temperature of 37°. If it has become coagulated it should be smoothed before culture and all clots removed.

The results usually can be read eighteen hours after culture. If the medium remains sterile, the fluid is not modified at all — it may darken a little, but remains homogeneous.

If typhus bacilli have grown, the medium becomes opaque, clotty and brownish. If the germs are paratyphic a great number of gas bubbles gather on the surface and others come up along the length of the glass. So much for the physician; but the bacteriologist needs know more. He will isolate the typhus bacilli to make sure there are no paratyphic bacilli, and if so to determine whether they are A or B, and also if any coli have developed in the hemoculture simulating a paratyphic. All this information is obtained in less than eighteen hours by a fresh growth of the hemoculture in lactosed lead gelose. In the research for the purity of the germs obtained in primary hemoculture in natural state, the bacilli of the typhic group are most often found in collections agglutinated by the patient's serum. When isolated they are mobile; after coloration by Gram, and fixation by alcohol they present little bacilli with rounded extremities, all Gram negative.

When the hemoculture is impure, a fresh isolation must be made and an identification test undertaken. Sometimes a simple culture on sloping gelose will eliminate the foreign germ. These verifying operations and others could be avoided by raising the initial blood by venous puncture.

In differentiating the paratyphics A and B from the coli bacillus in lactosed lead, one reads results as follows : Medium without gas and not darkened — paratyphic A. Medium without gas but darkened — paratyphic B. Medium with gas bubbles — coli bacillus. When the tubes are examined the microbian layer must be seen in profile.

